# Bis(1-benzyl-3-methyl­imidazolium-κ*C* ^2^)mercury(II) bis­(hexa­fluoridophosphate)

**DOI:** 10.1107/S1600536811032235

**Published:** 2011-08-17

**Authors:** Rosenani A. Haque, Abbas Washeel Salman, Madhukar Hemamalini, Hoong-Kun Fun

**Affiliations:** a School of Chemical Sciences, Universiti Sains Malaysia, 11800 USM, Penang, Malaysia; b X-ray Crystallography Unit, School of Physics, Universiti Sains Malaysia, 11800 USM, Penang, Malaysia

## Abstract

The asymmetric unit of the title complex, [Hg(C_11_H_12_N_2_)_2_](PF_6_)_2_, consists of one bis­(1-benzyl-3-methyl­imidazolium)mercury(II) cation, one half of the cation and an additional Hg^II^ atom, which lies on an inversion centre, and three hexa­fluorido­phosphate anions. The Hg^II^ atoms exist in a linear coordination geometry [C—Hg—C = 178.9 (2) and 180°] formed by two carbene C atoms from the imidazole rings. In the crystal, the cations and anions are connected *via* C—H⋯F hydrogen bonds, forming a three-dimensional network.

## Related literature

For details of *N*-heterocyclic carbenes, see: Herrmann (2002 ▶); Arduengo *et al.* (1991 ▶); Herrmann *et al.* (1998 ▶); McGuinness *et al.* (1999 ▶); Wanzlick & Schönherr (1968 ▶). For the stability of the temperature controller used in the data collection, see: Cosier & Glazer (1986 ▶).
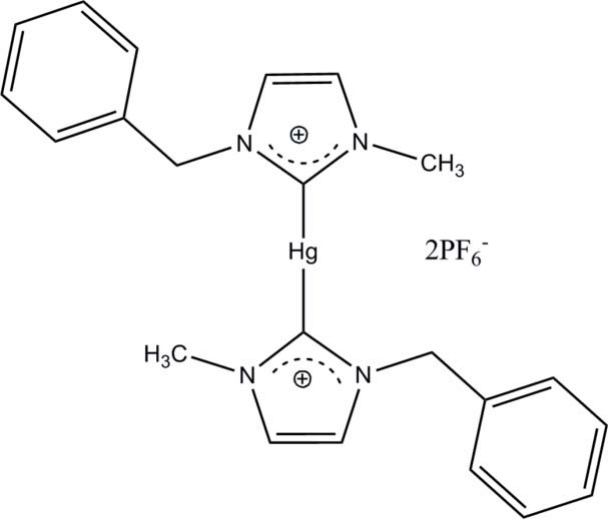


## Experimental

### 

#### Crystal data

[Hg(C_11_H_12_N_2_)_2_](PF_6_)_2_
*M* *_r_* = 834.98
Monoclinic, 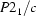

*a* = 15.1260 (17) Å
*b* = 10.3044 (11) Å
*c* = 26.398 (3) Å
β = 102.275 (2)°
*V* = 4020.5 (8) Å^3^
*Z* = 6
Mo *K*α radiation
μ = 5.97 mm^−1^
*T* = 100 K
0.34 × 0.32 × 0.05 mm

#### Data collection

Bruker APEXII DUO CCD area-detector diffractometer
Absorption correction: multi-scan (*SADABS*; Bruker, 2009 ▶) *T*_min_ = 0.233, *T*_max_ = 0.751
23876 measured reflections
7062 independent reflections
5985 reflections with *I* > 2σ(*I*)
*R*_int_ = 0.046

#### Refinement

*R*[*F* ^2^ > 2σ(*F* ^2^)] = 0.032
*wR*(*F* ^2^) = 0.087
*S* = 1.06
7062 reflections
559 parameters
H-atom parameters constrained
Δρ_max_ = 1.71 e Å^−3^
Δρ_min_ = −2.05 e Å^−3^

### 

Data collection: *APEX2* (Bruker, 2009 ▶); cell refinement: *SAINT* (Bruker, 2009 ▶); data reduction: *SAINT*; program(s) used to solve structure: *SHELXTL* (Sheldrick, 2008 ▶); program(s) used to refine structure: *SHELXTL*; molecular graphics: *SHELXTL*; software used to prepare material for publication: *SHELXTL* and *PLATON* (Spek, 2009 ▶).

## Supplementary Material

Crystal structure: contains datablock(s) global, I. DOI: 10.1107/S1600536811032235/is2765sup1.cif

Structure factors: contains datablock(s) I. DOI: 10.1107/S1600536811032235/is2765Isup2.hkl

Additional supplementary materials:  crystallographic information; 3D view; checkCIF report

## Figures and Tables

**Table 1:** Hydrogen-bond geometry (Å, °)

*D*—H⋯*A*	*D*—H	H⋯*A*	*D*⋯*A*	*D*—H⋯*A*
C10—H10*A*⋯F15^i^	0.93	2.32	3.240 (7)	171
C11—H11*C*⋯F6^ii^	0.96	2.55	3.375 (7)	144
C13—H13*A*⋯F7^i^	0.93	2.43	3.355 (7)	175
C18—H18*A*⋯F5^ii^	0.97	2.50	3.282 (6)	138
C18—H18*B*⋯F13^ii^	0.97	2.45	3.111 (6)	125
C21—H21*A*⋯F12^iii^	0.93	2.51	3.351 (6)	150
C29—H29*B*⋯F17^iv^	0.97	2.48	3.125 (7)	123
C31—H31*A*⋯F11^iv^	0.93	2.43	3.271 (6)	150

Symmetry codes: (i) 
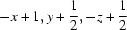
; (ii) 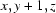; (iii) 
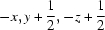
; (iv) 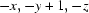.

**Fig. 1.**
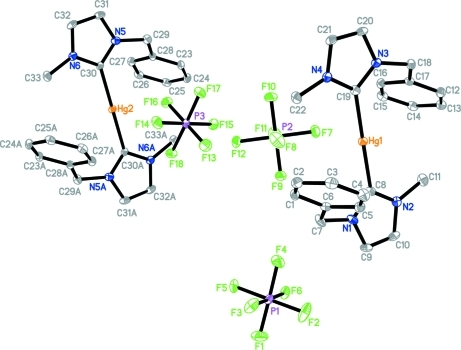
The asymmetric unit of the title compound, showing 30% probability displacement ellipsoids and the atom-numbering scheme. N5A–N6A/C23A–C32A are generated by the symmetry code -x, -y, -z (H atoms are omitted for clarity).

**Fig. 2.**
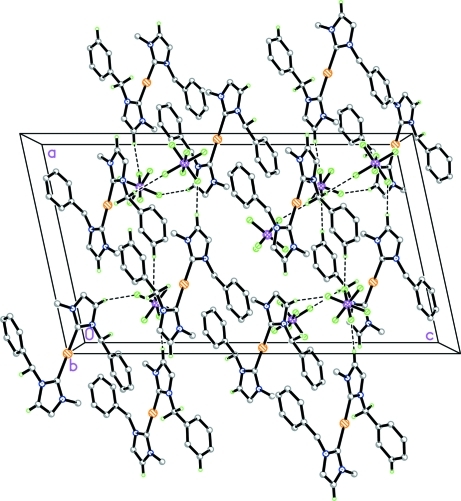
The crystal packing of the title compound, showing hydrogen-bonded (dashed lines) network.

## supplementary crystallographic information

### Comment

In the last two decades, *N*-heterocyclic carbene (NHC) ligands have emerged as useful and versatile ligands in organometallic chemistry (Herrmann, 2002). The chemistry of NHCs attracted much attention after the isolation of the first stable, crystalline free carbene (Arduengo *et al.*, 1991) which was [1,3-bis(adamantly)imidazole-2-ylidene]. Carbene ligands have some similarities to phosphine ligands, but metal- carbene complexes are often more stable than similar metal phosphine complexes (Herrmann *et al.*, 1998; McGuinness *et al.*, 1999). The first mercury(II)-NHC complex was prepared by Wanzlick and Schönherr (1968) *via* direct reaction of an imidazolium salt with mercury(II) acetate. However, in spite of being the earliest example of NHC-metal complexes prepared, NHC-mercury complexes have received little attention compared with other metals. Similarly, their applications have not been widely explored.

The asymmetric unit of title complex (I) consists of one bis(1-benzyl-3- methylimidazolium)mercury(II) cation, a half of the (1-benzyl-3-methyl imidazolium)mercury(II) cation (which lies on an inversion centre) and three hexafluorophosphate anions as shown in Fig. 1. The Hg^II^ atom exists in a linear coordination geometry formed by two C atoms from the imidazole rings. The bond distances of Hg1–C8 = 2.070 (5) Å; Hg1–C19 = 2.073 (5) Å and Hg2–C30 = 2.070 (5) Å.  The distorted octahedral geometry of phosphate ion has typical P–F distances [1.578 (4)–1.610 (3) Å] and F—P—F angles [88.37 (19)–179.4 (2)°]. All bond lengths and bond angles in (I) are in the range of expected values.

In the crystal structure (Fig. 2), ions are connected by C10—H10A···F15; C13—H13A···F7; C18—H18A···F5; C18—H18B···F13; C21—H21A···F12; C29—H29B···F17 and C31—H31A···F11 hydrogen bonds (Table 1), forming a three-dimensional network.

### Experimental

Hg(OAc)_2_ (0.35 g, 1.09 mmol) was added to a solution of 1-benzyl-3-methylimidazolium hexafluorophosphate (0.6 g, 1.88) in 40 ml of acetonitrile. The mixture was refluxed at 353–363 K for 18 h to give a clear solution. The solvent was removed under reduced pressure to afford a white solid. The white solid was collected, washed with distilled water (3 × 5 ml) and recrystallized from acetonitrile. Yield: 62.4 %, m.p. = 540–543 °C. Crystal suitable for X-ray analysis was obtained by slow diffusion of diethyl ether into solution of the complex in acetonitrile.

### Refinement

All hydrogen atoms were positioned geometrically (C—H = 0.93–0.97 Å) and were refined using a riding model, with *U*_iso_(H) = 1.2 or 1.5*U*_eq_(C). A rotating group model was applied to the methyl groups. The highest residual electron density peak is located at 1.30 Å from C8 and the deepest hole 0.96 Å located at from Hg2.

### Crystal data

**Table:**

[Hg(C_11_H_12_N_2_)_2_](PF_6_)_2_	*F*(000) = 2412
*M**_r_* = 834.98	*D*_x_ = 2.069 Mg m^−^^3^
Monoclinic, *P*2_1_/*c*	Mo *K*α radiation, λ = 0.71073 Å
Hall symbol: -P 2ybc	Cell parameters from 9967 reflections
*a* = 15.1260 (17) Å	θ = 2.8–29.9°
*b* = 10.3044 (11) Å	µ = 5.97 mm^−^^1^
*c* = 26.398 (3) Å	*T* = 100 K
β = 102.275 (2)°	Plate, colourless
*V* = 4020.5 (8) Å^3^	0.34 × 0.32 × 0.05 mm
*Z* = 6	

### Data collection

**Table:**

Bruker APEXII DUO CCD area-detector diffractometer	7062 independent reflections
Radiation source: fine-focus sealed tube	5985 reflections with *I* > 2σ(*I*)
graphite	*R*_int_ = 0.046
φ and ω scans	θ_max_ = 25.0°, θ_min_ = 1.9°
Absorption correction: multi-scan (*SADABS*; Bruker, 2009)	*h* = −17→17
*T*_min_ = 0.233, *T*_max_ = 0.751	*k* = −12→12
23876 measured reflections	*l* = −31→31

### Refinement

**Table:**

Refinement on *F*^2^	Primary atom site location: structure-invariant direct methods
Least-squares matrix: full	Secondary atom site location: difference Fourier map
*R*[*F*^2^ > 2σ(*F*^2^)] = 0.032	Hydrogen site location: inferred from neighbouring sites
*wR*(*F*^2^) = 0.087	H-atom parameters constrained
*S* = 1.06	*w* = 1/[σ^2^(*F*_o_^2^) + (0.0446*P*)^2^ + 4.1101*P*] where *P* = (*F*_o_^2^ + 2*F*_c_^2^)/3
7062 reflections	(Δ/σ)_max_ = 0.002
559 parameters	Δρ_max_ = 1.71 e Å^−^^3^
0 restraints	Δρ_min_ = −2.05 e Å^−^^3^

### Special details

**Table:**

Experimental. The crystal was placed in the cold stream of an Oxford Cryosystems Cobra open-flow nitrogen cryostat (Cosier & Glazer, 1986) operating at 100.0 (1) K.
Geometry. All s.u.'s (except the s.u. in the dihedral angle between two l.s. planes) are estimated using the full covariance matrix. The cell s.u.'s are taken into account individually in the estimation of s.u.'s in distances, angles and torsion angles; correlations between s.u.'s in cell parameters are only used when they are defined by crystal symmetry. An approximate (isotropic) treatment of cell s.u.'s is used for estimating s.u.'s involving l.s. planes.
Refinement. Refinement of F^2^ against ALL reflections. The weighted R-factor wR and goodness of fit S are based on F^2^, conventional R-factors R are based on F, with F set to zero for negative F^2^. The threshold expression of F^2^ > 2σ(F^2^) is used only for calculating R-factors(gt) etc. and is not relevant to the choice of reflections for refinement. R-factors based on F^2^ are statistically about twice as large as those based on F, and R- factors based on ALL data will be even larger.

### Fractional atomic coordinates and isotropic or equivalent isotropic displacement parameters (Å^2^)

**Table:**

	*x*	*y*	*z*	*U*_iso_*/*U*_eq_	
Hg1	0.315207 (14)	0.97602 (2)	0.334192 (7)	0.01976 (8)	
N1	0.4304 (3)	0.7691 (4)	0.40062 (17)	0.0236 (10)	
N2	0.5155 (3)	0.8950 (4)	0.36710 (16)	0.0224 (10)	
N3	0.2029 (3)	1.1919 (4)	0.27283 (16)	0.0202 (9)	
N4	0.1147 (3)	1.0569 (4)	0.30011 (16)	0.0212 (10)	
C1	0.2034 (4)	0.7697 (6)	0.4355 (2)	0.0257 (12)	
H1A	0.1790	0.6958	0.4176	0.031*	
C2	0.1494 (4)	0.8480 (6)	0.4601 (2)	0.0274 (13)	
H2A	0.0895	0.8256	0.4589	0.033*	
C3	0.1852 (4)	0.9572 (6)	0.4857 (2)	0.0286 (13)	
H3A	0.1496	1.0086	0.5024	0.034*	
C4	0.2739 (4)	0.9921 (6)	0.4872 (2)	0.0300 (14)	
H4A	0.2972	1.0678	0.5040	0.036*	
C5	0.3283 (4)	0.9140 (5)	0.4635 (2)	0.0242 (12)	
H5A	0.3882	0.9369	0.4649	0.029*	
C6	0.2933 (3)	0.8026 (5)	0.43784 (19)	0.0217 (11)	
C7	0.3504 (4)	0.7083 (6)	0.4139 (2)	0.0297 (13)	
H7A	0.3699	0.6379	0.4380	0.036*	
H7B	0.3132	0.6714	0.3827	0.036*	
C8	0.4286 (3)	0.8703 (5)	0.3685 (2)	0.0219 (11)	
C9	0.5174 (4)	0.7287 (6)	0.4192 (2)	0.0274 (13)	
H9A	0.5360	0.6602	0.4419	0.033*	
C10	0.5708 (4)	0.8078 (6)	0.3981 (2)	0.0284 (13)	
H10A	0.6336	0.8039	0.4035	0.034*	
C11	0.5465 (4)	0.9991 (6)	0.3372 (2)	0.0317 (14)	
H11A	0.4976	1.0259	0.3097	0.048*	
H11B	0.5956	0.9680	0.3227	0.048*	
H11C	0.5666	1.0715	0.3595	0.048*	
C12	0.4378 (4)	1.2409 (6)	0.2515 (2)	0.0265 (12)	
H12A	0.4532	1.3140	0.2722	0.032*	
C13	0.5017 (4)	1.1815 (6)	0.2282 (2)	0.0284 (13)	
H13A	0.5599	1.2152	0.2332	0.034*	
C14	0.4788 (4)	1.0730 (6)	0.1976 (2)	0.0269 (13)	
H14A	0.5214	1.0349	0.1815	0.032*	
C15	0.3924 (4)	1.0201 (5)	0.1908 (2)	0.0278 (13)	
H15A	0.3775	0.9453	0.1711	0.033*	
C16	0.3284 (3)	1.0807 (5)	0.21380 (19)	0.0221 (12)	
H16A	0.2702	1.0466	0.2088	0.027*	
C17	0.3499 (3)	1.1894 (5)	0.24355 (19)	0.0202 (11)	
C18	0.2829 (3)	1.2660 (5)	0.2668 (2)	0.0224 (12)	
H18A	0.3134	1.2979	0.3006	0.027*	
H18B	0.2631	1.3407	0.2450	0.027*	
C19	0.2020 (3)	1.0841 (5)	0.30121 (19)	0.0207 (11)	
C20	0.1155 (3)	1.2331 (6)	0.2541 (2)	0.0249 (12)	
H20A	0.0978	1.3053	0.2332	0.030*	
C21	0.0606 (3)	1.1502 (6)	0.2714 (2)	0.0273 (13)	
H21A	−0.0022	1.1547	0.2653	0.033*	
C22	0.0795 (4)	0.9499 (6)	0.3266 (2)	0.0300 (13)	
H22A	0.1277	0.9134	0.3522	0.045*	
H22B	0.0548	0.8843	0.3018	0.045*	
H22C	0.0331	0.9818	0.3431	0.045*	
P1	0.45866 (9)	0.30179 (15)	0.42352 (5)	0.0234 (3)	
F1	0.5096 (3)	0.1820 (4)	0.45364 (15)	0.0601 (12)	
F2	0.5246 (2)	0.3987 (5)	0.46037 (15)	0.0600 (13)	
F3	0.3896 (2)	0.3037 (4)	0.46149 (12)	0.0484 (11)	
F4	0.4061 (2)	0.4205 (3)	0.39212 (14)	0.0442 (9)	
F5	0.3921 (2)	0.2045 (3)	0.38564 (13)	0.0337 (8)	
F6	0.5263 (2)	0.2995 (3)	0.38445 (12)	0.0330 (8)	
P2	0.21906 (9)	0.66755 (14)	0.24439 (5)	0.0218 (3)	
F7	0.2844 (2)	0.7903 (3)	0.24821 (13)	0.0356 (8)	
F8	0.1816 (3)	0.7220 (4)	0.29269 (14)	0.0482 (10)	
F9	0.2952 (2)	0.5900 (3)	0.28381 (13)	0.0348 (8)	
F10	0.1427 (2)	0.7453 (4)	0.20554 (15)	0.0467 (10)	
F11	0.2578 (2)	0.6126 (4)	0.19728 (12)	0.0407 (9)	
F12	0.1528 (2)	0.5457 (3)	0.24149 (13)	0.0366 (8)	
Hg2	0.0000	0.0000	0.0000	0.01912 (9)	
N5	−0.1234 (3)	0.1955 (5)	−0.06934 (16)	0.0235 (10)	
N6	−0.2023 (3)	0.0628 (5)	−0.03454 (16)	0.0221 (10)	
C23	0.1087 (3)	0.2497 (5)	−0.09363 (19)	0.0243 (12)	
H23A	0.1211	0.3283	−0.0763	0.029*	
C24	0.1761 (4)	0.1875 (6)	−0.1134 (2)	0.0298 (14)	
H24A	0.2332	0.2245	−0.1091	0.036*	
C25	0.1581 (4)	0.0723 (6)	−0.1391 (2)	0.0271 (13)	
H25A	0.2028	0.0315	−0.1526	0.033*	
C26	0.0732 (4)	0.0163 (5)	−0.1451 (2)	0.0275 (13)	
H26A	0.0613	−0.0627	−0.1622	0.033*	
C27	0.0054 (4)	0.0778 (6)	−0.12544 (19)	0.0245 (12)	
H27A	−0.0516	0.0402	−0.1297	0.029*	
C28	0.0231 (3)	0.1943 (5)	−0.09981 (19)	0.0223 (12)	
C29	−0.0482 (4)	0.2735 (6)	−0.0805 (2)	0.0265 (12)	
H29A	−0.0193	0.3191	−0.0492	0.032*	
H29B	−0.0727	0.3381	−0.1064	0.032*	
C30	−0.1169 (3)	0.0956 (5)	−0.03602 (19)	0.0194 (11)	
C31	−0.2122 (4)	0.2274 (6)	−0.0878 (2)	0.0278 (13)	
H31A	−0.2341	0.2938	−0.1109	0.033*	
C32	−0.2616 (4)	0.1437 (6)	−0.0659 (2)	0.0288 (13)	
H32A	−0.3244	0.1414	−0.0712	0.035*	
C33	−0.2284 (4)	−0.0431 (6)	−0.0037 (2)	0.0283 (13)	
H33A	−0.1774	−0.0675	0.0230	0.042*	
H33B	−0.2478	−0.1164	−0.0258	0.042*	
H33C	−0.2770	−0.0149	0.0118	0.042*	
P3	0.12543 (9)	0.28634 (14)	0.09916 (5)	0.0229 (3)	
F13	0.1781 (3)	0.3315 (4)	0.15507 (13)	0.0482 (10)	
F14	0.0398 (2)	0.2474 (4)	0.12232 (14)	0.0420 (9)	
F15	0.2103 (2)	0.3240 (4)	0.07545 (13)	0.0426 (9)	
F16	0.0722 (2)	0.2397 (3)	0.04238 (12)	0.0351 (8)	
F17	0.0841 (2)	0.4277 (3)	0.08649 (13)	0.0371 (8)	
F18	0.1647 (2)	0.1422 (3)	0.11060 (12)	0.0348 (8)	

### Atomic displacement parameters (Å^2^)

**Table:**

	*U*^11^	*U*^22^	*U*^33^	*U*^12^	*U*^13^	*U*^23^
Hg1	0.01747 (12)	0.02072 (13)	0.02189 (12)	0.00238 (8)	0.00597 (8)	0.00041 (8)
N1	0.029 (3)	0.018 (2)	0.027 (2)	0.008 (2)	0.013 (2)	0.0043 (19)
N2	0.022 (2)	0.022 (2)	0.025 (2)	0.002 (2)	0.0088 (19)	−0.0036 (19)
N3	0.015 (2)	0.023 (2)	0.025 (2)	0.0042 (19)	0.0085 (18)	0.0001 (19)
N4	0.017 (2)	0.025 (2)	0.024 (2)	0.000 (2)	0.0084 (18)	0.000 (2)
C1	0.027 (3)	0.026 (3)	0.024 (3)	−0.008 (2)	0.005 (2)	0.003 (2)
C2	0.023 (3)	0.036 (3)	0.024 (3)	−0.002 (3)	0.008 (2)	0.006 (3)
C3	0.028 (3)	0.035 (3)	0.028 (3)	0.007 (3)	0.015 (2)	0.005 (3)
C4	0.034 (3)	0.028 (3)	0.031 (3)	0.001 (3)	0.012 (3)	−0.004 (2)
C5	0.021 (3)	0.028 (3)	0.025 (3)	0.001 (2)	0.007 (2)	0.001 (2)
C6	0.022 (3)	0.023 (3)	0.020 (3)	0.003 (2)	0.004 (2)	0.003 (2)
C7	0.033 (3)	0.020 (3)	0.042 (3)	0.001 (3)	0.022 (3)	0.003 (3)
C8	0.023 (3)	0.019 (3)	0.025 (3)	0.004 (2)	0.009 (2)	0.002 (2)
C9	0.029 (3)	0.029 (3)	0.024 (3)	0.013 (3)	0.005 (2)	0.004 (2)
C10	0.019 (3)	0.038 (4)	0.026 (3)	0.009 (3)	−0.001 (2)	−0.003 (3)
C11	0.028 (3)	0.032 (3)	0.040 (4)	−0.001 (3)	0.018 (3)	0.000 (3)
C12	0.023 (3)	0.027 (3)	0.030 (3)	−0.002 (2)	0.007 (2)	0.002 (2)
C13	0.016 (3)	0.034 (3)	0.038 (3)	0.000 (2)	0.011 (2)	0.012 (3)
C14	0.026 (3)	0.028 (3)	0.031 (3)	0.007 (3)	0.016 (2)	0.008 (2)
C15	0.037 (3)	0.025 (3)	0.023 (3)	0.004 (3)	0.012 (3)	0.000 (2)
C16	0.018 (3)	0.026 (3)	0.025 (3)	−0.004 (2)	0.009 (2)	0.002 (2)
C17	0.016 (3)	0.022 (3)	0.024 (3)	0.005 (2)	0.009 (2)	0.005 (2)
C18	0.021 (3)	0.022 (3)	0.026 (3)	−0.002 (2)	0.008 (2)	−0.002 (2)
C19	0.018 (3)	0.024 (3)	0.023 (3)	0.003 (2)	0.012 (2)	0.001 (2)
C20	0.018 (3)	0.029 (3)	0.027 (3)	0.008 (2)	0.003 (2)	0.001 (2)
C21	0.015 (3)	0.035 (3)	0.031 (3)	0.003 (2)	0.004 (2)	0.001 (3)
C22	0.023 (3)	0.031 (3)	0.040 (3)	−0.002 (3)	0.016 (3)	0.007 (3)
P1	0.0168 (7)	0.0314 (8)	0.0225 (7)	−0.0002 (6)	0.0054 (6)	−0.0045 (6)
F1	0.044 (2)	0.074 (3)	0.061 (3)	0.021 (2)	0.009 (2)	0.033 (2)
F2	0.036 (2)	0.088 (3)	0.058 (2)	−0.017 (2)	0.0141 (19)	−0.044 (2)
F3	0.0268 (18)	0.094 (3)	0.0278 (18)	−0.002 (2)	0.0140 (15)	−0.008 (2)
F4	0.044 (2)	0.028 (2)	0.063 (2)	0.0109 (17)	0.0169 (19)	0.0044 (18)
F5	0.0269 (17)	0.0280 (19)	0.048 (2)	−0.0072 (15)	0.0110 (15)	−0.0115 (16)
F6	0.0233 (17)	0.046 (2)	0.0332 (18)	−0.0088 (16)	0.0142 (14)	−0.0085 (16)
P2	0.0182 (7)	0.0220 (7)	0.0261 (7)	−0.0002 (6)	0.0068 (6)	−0.0002 (6)
F7	0.0307 (18)	0.0258 (19)	0.052 (2)	−0.0038 (15)	0.0115 (16)	0.0000 (16)
F8	0.066 (3)	0.038 (2)	0.053 (2)	0.009 (2)	0.041 (2)	−0.0030 (18)
F9	0.0252 (17)	0.035 (2)	0.043 (2)	0.0026 (15)	0.0034 (15)	0.0113 (16)
F10	0.0290 (19)	0.044 (2)	0.063 (2)	0.0097 (17)	0.0005 (17)	0.0187 (19)
F11	0.054 (2)	0.040 (2)	0.0351 (19)	−0.0009 (18)	0.0250 (17)	−0.0065 (16)
F12	0.0218 (17)	0.0309 (19)	0.056 (2)	−0.0058 (15)	0.0061 (15)	0.0047 (17)
Hg2	0.01532 (15)	0.02267 (16)	0.02001 (15)	0.00282 (11)	0.00519 (11)	0.00063 (11)
N5	0.023 (2)	0.026 (3)	0.022 (2)	0.007 (2)	0.0074 (19)	0.001 (2)
N6	0.019 (2)	0.028 (3)	0.021 (2)	0.001 (2)	0.0081 (18)	0.000 (2)
C23	0.026 (3)	0.023 (3)	0.024 (3)	−0.006 (2)	0.006 (2)	0.000 (2)
C24	0.020 (3)	0.041 (4)	0.032 (3)	−0.006 (3)	0.012 (2)	0.010 (3)
C25	0.031 (3)	0.030 (3)	0.025 (3)	0.008 (3)	0.015 (2)	0.007 (2)
C26	0.035 (3)	0.024 (3)	0.027 (3)	0.007 (3)	0.015 (3)	0.002 (2)
C27	0.024 (3)	0.029 (3)	0.023 (3)	−0.001 (2)	0.012 (2)	0.001 (2)
C28	0.022 (3)	0.023 (3)	0.023 (3)	−0.001 (2)	0.007 (2)	0.003 (2)
C29	0.028 (3)	0.026 (3)	0.029 (3)	0.001 (3)	0.012 (2)	−0.001 (2)
C30	0.014 (3)	0.023 (3)	0.023 (3)	0.001 (2)	0.008 (2)	0.000 (2)
C31	0.024 (3)	0.040 (4)	0.020 (3)	0.013 (3)	0.005 (2)	0.002 (2)
C32	0.016 (3)	0.040 (4)	0.030 (3)	0.007 (3)	0.004 (2)	−0.004 (3)
C33	0.024 (3)	0.030 (3)	0.036 (3)	0.000 (3)	0.017 (3)	0.002 (3)
P3	0.0212 (7)	0.0235 (8)	0.0252 (7)	−0.0024 (6)	0.0078 (6)	−0.0020 (6)
F13	0.064 (2)	0.045 (2)	0.0311 (19)	−0.007 (2)	−0.0009 (18)	−0.0066 (17)
F14	0.038 (2)	0.040 (2)	0.057 (2)	0.0023 (17)	0.0284 (18)	0.0121 (18)
F15	0.0201 (17)	0.057 (3)	0.054 (2)	−0.0034 (17)	0.0155 (16)	0.0187 (19)
F16	0.0354 (19)	0.035 (2)	0.0321 (18)	0.0034 (16)	0.0011 (15)	−0.0069 (15)
F17	0.046 (2)	0.0260 (19)	0.042 (2)	0.0047 (17)	0.0158 (17)	0.0026 (16)
F18	0.0357 (19)	0.0311 (19)	0.0369 (19)	0.0091 (16)	0.0061 (15)	0.0029 (15)

### Geometric parameters (Å, °)

**Table:**

Hg1—C8	2.070 (5)	C21—H21A	0.9300
Hg1—C19	2.073 (5)	C22—H22A	0.9600
N1—C8	1.341 (7)	C22—H22B	0.9600
N1—C9	1.367 (7)	C22—H22C	0.9600
N1—C7	1.470 (7)	P1—F1	1.578 (4)
N2—C8	1.348 (6)	P1—F2	1.589 (4)
N2—C10	1.373 (7)	P1—F4	1.592 (4)
N2—C11	1.467 (7)	P1—F3	1.594 (3)
N3—C19	1.341 (7)	P1—F6	1.600 (3)
N3—C20	1.377 (6)	P1—F5	1.610 (3)
N3—C18	1.467 (6)	P2—F11	1.588 (3)
N4—C19	1.344 (6)	P2—F10	1.588 (4)
N4—C21	1.380 (7)	P2—F9	1.593 (3)
N4—C22	1.464 (7)	P2—F7	1.595 (3)
C1—C6	1.391 (7)	P2—F12	1.598 (3)
C1—C2	1.401 (8)	P2—F8	1.603 (3)
C1—H1A	0.9300	Hg2—C30^i^	2.070 (5)
C2—C3	1.365 (8)	Hg2—C30	2.070 (5)
C2—H2A	0.9300	N5—C30	1.344 (7)
C3—C4	1.381 (8)	N5—C31	1.368 (7)
C3—H3A	0.9300	N5—C29	1.472 (7)
C4—C5	1.392 (8)	N6—C30	1.344 (6)
C4—H4A	0.9300	N6—C32	1.368 (7)
C5—C6	1.379 (8)	N6—C33	1.465 (7)
C5—H5A	0.9300	C23—C28	1.393 (7)
C6—C7	1.523 (7)	C23—C24	1.396 (7)
C7—H7A	0.9700	C23—H23A	0.9300
C7—H7B	0.9700	C24—C25	1.365 (8)
C9—C10	1.350 (8)	C24—H24A	0.9300
C9—H9A	0.9300	C25—C26	1.387 (8)
C10—H10A	0.9300	C25—H25A	0.9300
C11—H11A	0.9600	C26—C27	1.395 (7)
C11—H11B	0.9600	C26—H26A	0.9300
C11—H11C	0.9600	C27—C28	1.376 (8)
C12—C13	1.393 (7)	C27—H27A	0.9300
C12—C17	1.405 (7)	C28—C29	1.524 (7)
C12—H12A	0.9300	C29—H29A	0.9700
C13—C14	1.379 (8)	C29—H29B	0.9700
C13—H13A	0.9300	C31—C32	1.350 (8)
C14—C15	1.392 (8)	C31—H31A	0.9300
C14—H14A	0.9300	C32—H32A	0.9300
C15—C16	1.396 (7)	C33—H33A	0.9600
C15—H15A	0.9300	C33—H33B	0.9600
C16—C17	1.367 (7)	C33—H33C	0.9600
C16—H16A	0.9300	P3—F13	1.591 (4)
C17—C18	1.514 (7)	P3—F15	1.591 (3)
C18—H18A	0.9700	P3—F17	1.592 (4)
C18—H18B	0.9700	P3—F14	1.597 (3)
C20—C21	1.337 (7)	P3—F18	1.604 (3)
C20—H20A	0.9300	P3—F16	1.616 (3)
			
C8—Hg1—C19	178.9 (2)	F1—P1—F2	90.4 (3)
C8—N1—C9	110.7 (5)	F1—P1—F4	178.7 (3)
C8—N1—C7	125.1 (5)	F2—P1—F4	90.9 (2)
C9—N1—C7	124.3 (5)	F1—P1—F3	90.5 (2)
C8—N2—C10	109.6 (4)	F2—P1—F3	91.0 (2)
C8—N2—C11	125.3 (5)	F4—P1—F3	89.8 (2)
C10—N2—C11	125.1 (5)	F1—P1—F6	90.1 (2)
C19—N3—C20	109.5 (4)	F2—P1—F6	89.97 (19)
C19—N3—C18	126.7 (4)	F4—P1—F6	89.56 (19)
C20—N3—C18	123.5 (5)	F3—P1—F6	178.9 (2)
C19—N4—C21	109.3 (4)	F1—P1—F5	90.0 (2)
C19—N4—C22	127.0 (5)	F2—P1—F5	179.4 (2)
C21—N4—C22	123.6 (4)	F4—P1—F5	88.73 (19)
C6—C1—C2	120.0 (5)	F3—P1—F5	89.51 (19)
C6—C1—H1A	120.0	F6—P1—F5	89.53 (17)
C2—C1—H1A	120.0	F11—P2—F10	90.7 (2)
C3—C2—C1	119.7 (5)	F11—P2—F9	89.8 (2)
C3—C2—H2A	120.1	F10—P2—F9	179.4 (2)
C1—C2—H2A	120.1	F11—P2—F7	90.32 (19)
C2—C3—C4	120.6 (5)	F10—P2—F7	89.8 (2)
C2—C3—H3A	119.7	F9—P2—F7	90.26 (19)
C4—C3—H3A	119.7	F11—P2—F12	90.8 (2)
C3—C4—C5	120.0 (6)	F10—P2—F12	90.18 (19)
C3—C4—H4A	120.0	F9—P2—F12	89.78 (19)
C5—C4—H4A	120.0	F7—P2—F12	178.9 (2)
C6—C5—C4	120.0 (5)	F11—P2—F8	178.9 (2)
C6—C5—H5A	120.0	F10—P2—F8	90.3 (2)
C4—C5—H5A	120.0	F9—P2—F8	89.1 (2)
C5—C6—C1	119.6 (5)	F7—P2—F8	89.5 (2)
C5—C6—C7	122.8 (5)	F12—P2—F8	89.4 (2)
C1—C6—C7	117.5 (5)	C30^i^—Hg2—C30	180.0 (4)
N1—C7—C6	113.3 (5)	C30—N5—C31	110.4 (5)
N1—C7—H7A	108.9	C30—N5—C29	126.6 (4)
C6—C7—H7A	108.9	C31—N5—C29	122.6 (5)
N1—C7—H7B	108.9	C30—N6—C32	109.9 (4)
C6—C7—H7B	108.9	C30—N6—C33	125.3 (5)
H7A—C7—H7B	107.7	C32—N6—C33	124.8 (5)
N1—C8—N2	105.9 (5)	C28—C23—C24	120.2 (5)
N1—C8—Hg1	126.1 (4)	C28—C23—H23A	119.9
N2—C8—Hg1	127.7 (4)	C24—C23—H23A	119.9
C10—C9—N1	106.4 (5)	C25—C24—C23	120.1 (5)
C10—C9—H9A	126.8	C25—C24—H24A	120.0
N1—C9—H9A	126.8	C23—C24—H24A	120.0
C9—C10—N2	107.4 (5)	C24—C25—C26	120.0 (5)
C9—C10—H10A	126.3	C24—C25—H25A	120.0
N2—C10—H10A	126.3	C26—C25—H25A	120.0
N2—C11—H11A	109.5	C25—C26—C27	120.3 (5)
N2—C11—H11B	109.5	C25—C26—H26A	119.8
H11A—C11—H11B	109.5	C27—C26—H26A	119.8
N2—C11—H11C	109.5	C28—C27—C26	119.8 (5)
H11A—C11—H11C	109.5	C28—C27—H27A	120.1
H11B—C11—H11C	109.5	C26—C27—H27A	120.1
C13—C12—C17	119.4 (5)	C27—C28—C23	119.6 (5)
C13—C12—H12A	120.3	C27—C28—C29	123.6 (5)
C17—C12—H12A	120.3	C23—C28—C29	116.6 (5)
C14—C13—C12	120.2 (5)	N5—C29—C28	114.0 (5)
C14—C13—H13A	119.9	N5—C29—H29A	108.8
C12—C13—H13A	119.9	C28—C29—H29A	108.8
C13—C14—C15	120.3 (5)	N5—C29—H29B	108.8
C13—C14—H14A	119.8	C28—C29—H29B	108.8
C15—C14—H14A	119.8	H29A—C29—H29B	107.7
C14—C15—C16	119.2 (5)	N6—C30—N5	105.9 (4)
C14—C15—H15A	120.4	N6—C30—Hg2	126.9 (4)
C16—C15—H15A	120.4	N5—C30—Hg2	127.0 (4)
C17—C16—C15	121.0 (5)	C32—C31—N5	106.6 (5)
C17—C16—H16A	119.5	C32—C31—H31A	126.7
C15—C16—H16A	119.5	N5—C31—H31A	126.7
C16—C17—C12	119.8 (5)	C31—C32—N6	107.2 (5)
C16—C17—C18	124.2 (5)	C31—C32—H32A	126.4
C12—C17—C18	115.9 (5)	N6—C32—H32A	126.4
N3—C18—C17	114.3 (4)	N6—C33—H33A	109.5
N3—C18—H18A	108.7	N6—C33—H33B	109.5
C17—C18—H18A	108.7	H33A—C33—H33B	109.5
N3—C18—H18B	108.7	N6—C33—H33C	109.5
C17—C18—H18B	108.7	H33A—C33—H33C	109.5
H18A—C18—H18B	107.6	H33B—C33—H33C	109.5
N3—C19—N4	106.7 (5)	F13—P3—F15	90.4 (2)
N3—C19—Hg1	125.0 (4)	F13—P3—F17	91.4 (2)
N4—C19—Hg1	128.1 (4)	F15—P3—F17	90.44 (19)
C21—C20—N3	107.4 (5)	F13—P3—F14	90.3 (2)
C21—C20—H20A	126.3	F15—P3—F14	179.3 (2)
N3—C20—H20A	126.3	F17—P3—F14	89.77 (19)
C20—C21—N4	107.1 (4)	F13—P3—F18	90.2 (2)
C20—C21—H21A	126.4	F15—P3—F18	90.16 (19)
N4—C21—H21A	126.4	F17—P3—F18	178.3 (2)
N4—C22—H22A	109.5	F14—P3—F18	89.61 (19)
N4—C22—H22B	109.5	F13—P3—F16	179.7 (2)
H22A—C22—H22B	109.5	F15—P3—F16	89.57 (19)
N4—C22—H22C	109.5	F17—P3—F16	88.93 (19)
H22A—C22—H22C	109.5	F14—P3—F16	89.74 (19)
H22B—C22—H22C	109.5	F18—P3—F16	89.47 (18)
			
C6—C1—C2—C3	0.8 (8)	C18—N3—C19—N4	173.6 (4)
C1—C2—C3—C4	0.7 (9)	C20—N3—C19—Hg1	175.8 (4)
C2—C3—C4—C5	−1.6 (9)	C18—N3—C19—Hg1	−11.0 (7)
C3—C4—C5—C6	0.9 (9)	C21—N4—C19—N3	−1.1 (6)
C4—C5—C6—C1	0.6 (8)	C22—N4—C19—N3	−178.4 (5)
C4—C5—C6—C7	−176.1 (5)	C21—N4—C19—Hg1	−176.2 (4)
C2—C1—C6—C5	−1.5 (8)	C22—N4—C19—Hg1	6.5 (8)
C2—C1—C6—C7	175.5 (5)	C19—N3—C20—C21	0.4 (6)
C8—N1—C7—C6	−58.1 (7)	C18—N3—C20—C21	−173.1 (5)
C9—N1—C7—C6	122.6 (6)	N3—C20—C21—N4	−1.0 (6)
C5—C6—C7—N1	−24.6 (7)	C19—N4—C21—C20	1.3 (6)
C1—C6—C7—N1	158.5 (5)	C22—N4—C21—C20	178.7 (5)
C9—N1—C8—N2	−0.6 (6)	C28—C23—C24—C25	0.2 (8)
C7—N1—C8—N2	−179.9 (5)	C23—C24—C25—C26	−0.7 (8)
C9—N1—C8—Hg1	−175.3 (4)	C24—C25—C26—C27	0.8 (8)
C7—N1—C8—Hg1	5.4 (8)	C25—C26—C27—C28	−0.4 (8)
C10—N2—C8—N1	0.7 (6)	C26—C27—C28—C23	−0.1 (8)
C11—N2—C8—N1	−179.3 (5)	C26—C27—C28—C29	175.4 (5)
C10—N2—C8—Hg1	175.2 (4)	C24—C23—C28—C27	0.2 (8)
C11—N2—C8—Hg1	−4.7 (8)	C24—C23—C28—C29	−175.7 (5)
C8—N1—C9—C10	0.3 (6)	C30—N5—C29—C28	57.7 (7)
C7—N1—C9—C10	179.7 (5)	C31—N5—C29—C28	−130.7 (5)
N1—C9—C10—N2	0.1 (6)	C27—C28—C29—N5	26.5 (7)
C8—N2—C10—C9	−0.5 (6)	C23—C28—C29—N5	−157.8 (5)
C11—N2—C10—C9	179.5 (5)	C32—N6—C30—N5	−1.3 (6)
C17—C12—C13—C14	−0.2 (8)	C33—N6—C30—N5	179.1 (5)
C12—C13—C14—C15	−1.4 (8)	C32—N6—C30—Hg2	−175.8 (4)
C13—C14—C15—C16	2.1 (8)	C33—N6—C30—Hg2	4.5 (8)
C14—C15—C16—C17	−1.1 (8)	C31—N5—C30—N6	1.4 (6)
C15—C16—C17—C12	−0.4 (8)	C29—N5—C30—N6	173.9 (5)
C15—C16—C17—C18	175.7 (5)	C31—N5—C30—Hg2	175.9 (4)
C13—C12—C17—C16	1.1 (8)	C29—N5—C30—Hg2	−11.6 (8)
C13—C12—C17—C18	−175.4 (5)	C30—N5—C31—C32	−1.0 (6)
C19—N3—C18—C17	60.2 (7)	C29—N5—C31—C32	−173.8 (5)
C20—N3—C18—C17	−127.5 (5)	N5—C31—C32—N6	0.2 (6)
C16—C17—C18—N3	22.5 (7)	C30—N6—C32—C31	0.7 (6)
C12—C17—C18—N3	−161.2 (5)	C33—N6—C32—C31	−179.6 (5)
C20—N3—C19—N4	0.5 (6)		

Symmetry codes: (i) −*x*, −*y*, −*z*.

### Hydrogen-bond geometry (Å, °)

**Table:**

*D*—H···*A*	*D*—H	H···*A*	*D*···*A*	*D*—H···*A*
C10—H10A···F15^ii^	0.93	2.32	3.240 (7)	171
C11—H11C···F6^iii^	0.96	2.55	3.375 (7)	144
C13—H13A···F7^ii^	0.93	2.43	3.355 (7)	175
C18—H18A···F5^iii^	0.97	2.50	3.282 (6)	138
C18—H18B···F13^iii^	0.97	2.45	3.111 (6)	125
C21—H21A···F12^iv^	0.93	2.51	3.351 (6)	150
C29—H29B···F17^v^	0.97	2.48	3.125 (7)	123
C31—H31A···F11^v^	0.93	2.43	3.271 (6)	150

Symmetry codes: (ii) −*x*+1, *y*+1/2, −*z*+1/2; (iii) *x*, *y*+1, *z*; (iv) −*x*, *y*+1/2, −*z*+1/2; (v) −*x*, −*y*+1, −*z*.
